# A comparative study of COVID-19 point-of-care detection across human biofluids using MEMS-based near-infrared spectroscopy and machine learning

**DOI:** 10.1007/s10544-026-00806-0

**Published:** 2026-04-13

**Authors:** Ahmed Abdelkhalik, Mazen Erfan, Bassem Mortada, Mohamed Gaber, Moataz Bellah Abdelaleem, Hala Hafez, Samia A. Girgis, Ossama Mansour, Bassam Saadany, Yasser M. Sabry, Diaa Khalil

**Affiliations:** 1 Si-Ware Systems, 3 Khaled Ibn Al-Waleed Street, Heliopolis, Cairo, Egypt; 2 Faculty of Engineering, ECE Department, Ain Shams University, Cairo, Egypt; 3 Faculty of Medicine, Clinical Pathology Department, Ain Shams University, Cairo, Egypt; 4 Faculty of Medicine, Ear, Nose and Throat Department, Ain Shams University, Cairo, Egypt

**Keywords:** ATR, COVID-19, Human biofluids, MEMS FTIR spectrometer, NIR spectroscopy, PCR, Infectious disease

## Abstract

This study explores the use of near-infrared (NIR) spectroscopy in the 1.3–2.6 μm wavelength range, employing a handheld miniaturized microelectromechanical systems (MEMS)-based spectrometer for the rapid, non-invasive detection of COVID-19 from various biofluids. A total of 238 samples—including nasopharyngeal (NSP) swabs, nasal swabs, and saliva—from both COVID-19 positive and negative individuals are analysed. Machine learning algorithms process the spectral data to develop predictive models for the disease classification. Models based on a single biofluid achieve detection accuracies between 75% and 80%, while combining scans from multiple biofluids of the same individual improves accuracy to 88%. The study highlights trade-offs between sample accessibility and diagnostic performance. Overall, the findings demonstrate that NIR spectroscopy serves as a viable low-cost, portable, and rapid point-of-care (POC) solution, with strong potential for scalable mass screening—particularly in resource-limited settings.

## Introduction

The COVID-19 disease caused by the severe acute respiratory syndrome coronavirus 2 (SARS-CoV-2) virus was recognized as a global pandemic by the WHO on March 11, 2020 (Cucinotta and Vanelli 2020). Since then, it was associated with the deaths of approximately 14.9 million patients worldwide in 2020 and 2021; this includes deaths associated with COVID-19 directly or due to the pandemic’s impact on health systems and society. COVID-19 is a highly infectious disease with an average of 2 or 3 people infected by a single carrier before the symptoms appear. In 2022, the number of patients worldwide has declined and the disease is slowly becoming a seasonal disease like influenza. The COVID-19 virus is still causing seasonal outbreaks of newer variants, with the most recent variant being omicron KP.3. The guidelines of the WHO recommends the maintained surveillance of the virus spread and the scaling of the vaccines in order to overcome any dangerous emerging variants (WHO 2022).

In order to comply with the WHO guidelines in maintaining surveillance of the virus spread, the methods of detection of the SARS-CoV-2 virus need to become faster and more affordable, with the most recent products including ID NOW isothermal amplification technology which produces results within 15 min (Tu et al. 2021). However, the associated cost of the tests stands as a barrier to their use in mass screening. Antigen detection was also presented as a low-cost rapid solution, but it was only recommended in restricted use-cases WHO (2021) due to its narrow performance criteria. Other available methods, such as serology tests, suffer from a delay of about one week between infection and detectability. These methods are also generally limited to detecting severe cases, making them less suitable for identifying emerging variants. As a result, the WHO continues to recommend RT-PCR as the gold standard for detecting SARS-CoV-2 and its variants WHO (2021).

Lindner, et al. (2021) and Khiabani and Amirzade-Iranaq (2021) have recently shown the capability of different biological samples in the detection of COVID-19 through antigen based methods such as nasopharyngeal, oropharyngeal, nasal, and sputum, showing that a mix of nasopharyngeal and oropharyngeal swab sample has the highest sensitivity of almost 95%, with nasal and saliva swabs having almost 90% agreement. Saliva and nasal samples offer an easier sampling procedure which reduces the requirement of trained nurses for sample extraction, and allows self-collection of samples. Another merit of using saliva and nasal samples is the availability of other biological materials in the samples which act as biomarkers that help in the detection of the virus.

The need for low-cost point-of-care testing methods has accelerated the evolution of biomedical sensors for viral detection and other health conditions. Biomedical sensors include electrochemical sensors (Biswas et al. 2022), plasmonic sensors (Wang et al. 2023), and Terahertz sensors (Rafighirani et al. 2025). Similarly, IR spectroscopy is seeing a significant surge of interest within the research community as a viable tool for low-cost point-of-care testing (Mayerhöfer et al. 2021).

Fourier Transform Infrared (FTIR) spectroscopy is a tool for analyzing material composition by measuring the interaction between infrared light and molecular components. It has been used in pharmaceutical analysis for many years (Fahelelbom et al. 2022). FTIR has been applied in the detection of various diseases and viruses including cancer (Su and Lee 2020), Leukemia (Sheng et al. 2013), HIV (Fadlelmoula et al. 2022), and hepatitis C (Roy et al. 2019) through blood. It is also used in detecting diseases through skin (Salem et al. 2021) and hair analysis (Al-Kelani and Buthelezi 2024), which makes FTIR spectroscopy one of the most versatile and affordable methods for health monitoring. This research aims to further extend FTIR spectroscopy use in biomedical testing by evaluating its performance in the analysis of different biofluids.

Wood, Bayden R., et al. (2021) recently demonstrated FTIR spectroscopy capability in the detection of COVID-19 in saliva samples, achieving 93% snsitivity and 82% secificity. In that study, samples were air-dried on a BaF_2_ window then placed on a benchtop spectrometer over a period of 15 min, which makes this method less suitable for POC mass screening due to time and equipment constraint. Other body fluids, such as blood plasma has also been investigated. Calvo-Gomez, Octavio, et al. (2022) reported FTIR spectroscopy capability of detecting COVID-19 with 94.55% sensitivity and 98.44% specificity in blood samples. A similar study for FTIR detection of the virus using blood plasma was conducted by Zhang, Liyang, et al. (2021), which achieved 83% sensitivity and 98% specificity. Moreover, the capability of using the same VTM sample in both FTIR and PCR analyses was previously investigated by Nogueira et al. (2021), and an accuracy of 78% was achieved. In the latter study, the samples were dried on an aluminum foil in laminar air for 2 h, a process that may reduce viral content and affect detection performance.

Infrared spectroscopy in the NIR region has the advantage of measuring liquid samples without any preparation steps and with a penetration depth in the millimeter range. The extended NIR region captures strong overtone signals of fundamental chemical bonds, including both the first and second overtones of C–H bonds (Eldin 2011). A high interaction length in liquids allows its use in the analysis of very dense fluids such as milk (Amr et al. 2018); this capability distinguishes NIR spectroscopy from other infrared methods—such as mid and far-infrared spectroscopy—which are typically limited to surface-level scanning.

Miniaturized microelectromechanical systems (MEMS) based FTIR spectrometers are novel instruments that have been employed in feed testing (Khater et al. 2024), environmental pollution monitoring (Fathy et al. 2023) and biomedical studies including skin analysis (Salem et al. 2021). Moreover, it has also shown its effectiveness in the analysis of biofluids (Othman et al. 2024), making it a very attractive low cost device for mass screening. However, MEMS FTIR spectroscopy scope of analysis is limited by the reduced capabilities of the device in terms of signal-to-noise ratio, spectral resolution and range of wavelengths detectable especially when using uncooled optical detector (Ghoname et al. 2019).

In this work, portable FT-NIR spectroscopy using a MEMS-based spectrometer (Erfan et al. 2016, Fathy et al. 2019, Sabry et al. 2017) is evaluated as a potential tool for affordable point of care mass screening of COVID-19. Taking advantage of the fast and low cost-scanning capability of MEMS FT-NIR spectrometer, this study analyses multiple biofluids—specifically saliva, nasal, and nasopharyngeal samples stored in VTM. The resulting spectra are processed using machine learning algorithms to identify spectral regions that correlate with the presence of the disease. This approach assesses the feasibility of MEMS FT-NIR spectroscopy for COVID-19 detection in the near-infrared region and compares the diagnostic performance across different biofluids.

## Methods

### Samples collection

The aim of this study is to investigate the usability of optical spectroscopy in the near-infrared region for the detection of the COVID-19 disease using different sampling methods. The most promising sampling schemes used are nasopharyngeal swabs, nasal swabs, and saliva samples, all of which have demonstrated high accuracy in SARS-CoV-2 detection in previous studies (Tsang et al. 2021).

Sample collection started at Ain Shams University hospitals laboratory with the approval of the Research Ethics Committee (FMASU REC) with Federal wide assurance No. 00017585. The research mainly focused on samples of patients from nearby isolation hospitals which were using the COVID-19 lab in the hospital for periodic PCR scans. Doctors in isolation hospitals were instructed to collect a nasal swab sample and a saliva sample in addition to the nasopharyngeal swab sample that was periodically takenfrom patients, and a written consent was taken from each patient before the sample collection.

Nasopharyngeal and nasal samples were each stored in 500 µL of guanidine-based viral transport medium (VTM) in airtight VTM transport tubes, with the goal of maximizing the virus concentration. Of this volume, 300 µL were allocated for PCR analysis, while the remaining 200 µL facilitated VTM extraction for evaluation using the NIR handheld spectrometer. As such, a VTM volume of 500 µL was determined to be optimal for this study. In contrast, saliva samples were collected in standard empty collection cups. Each participant was instructed to accumulate saliva in the mouth for one minute before spitting into the cup. No restrictions were placed on the time elapsed since the last meal, which may have introduced variability into the results. Additionally, no preservatives were added to the saliva samples to maintain the highest possible viral concentration. No preservatives were added as the samples were not intended for PCR or other polymerization-based reactions.

A total of 93 patients from the isolation hospitals participated in this study, including 53 individuals who tested positive and 40 individuals who tested negative for COVID-19. The reference method for determining infection status was the PCR test, which is considered the gold standard for SARS-CoV-2 detection. PCR testing was performed exclusively on nasopharyngeal VTM samples, which was reported to have 85% sensitivity and near perfect specificity (Gopaul et al. 2020). Viral loads in nasal and saliva samples were assumed to be proportional to those in nasopharyngeal samples, as supported by findings in previous studies (Lindner et al. 2021, Khiabani and Amirzade-Iranaq 2021).

Samples were transferred from the hospitals in sealed containers. Upon arrival, nasopharyngeal samples were tested using PCR within a few hours. All samples were scanned using the portable FT-NIR spectrometer within 12 h of arrival, which is within the acceptable timeframe to preserve viral integrity. During this period, samples were stored in air conditioned room temperature, which was approximately 25 °C, without refrigeration in order to avoid introducing variability that could result from temperature changes during cooling and reheating.

Due to the absence of volumetric saliva collection kits, a portion of the saliva samples experienced partial evaporation between collection and measurement. Additionally, some patients who consented to the study declined to provide saliva samples, which is likely due to discomfort or aversion, which may have been influenced by the clinical setting. This highlights a limitation of in-clinic saliva collection compared to the more comfortable option of self-collection at home.

### Portable scanning device and measurement procedure

**Fig. 1:**
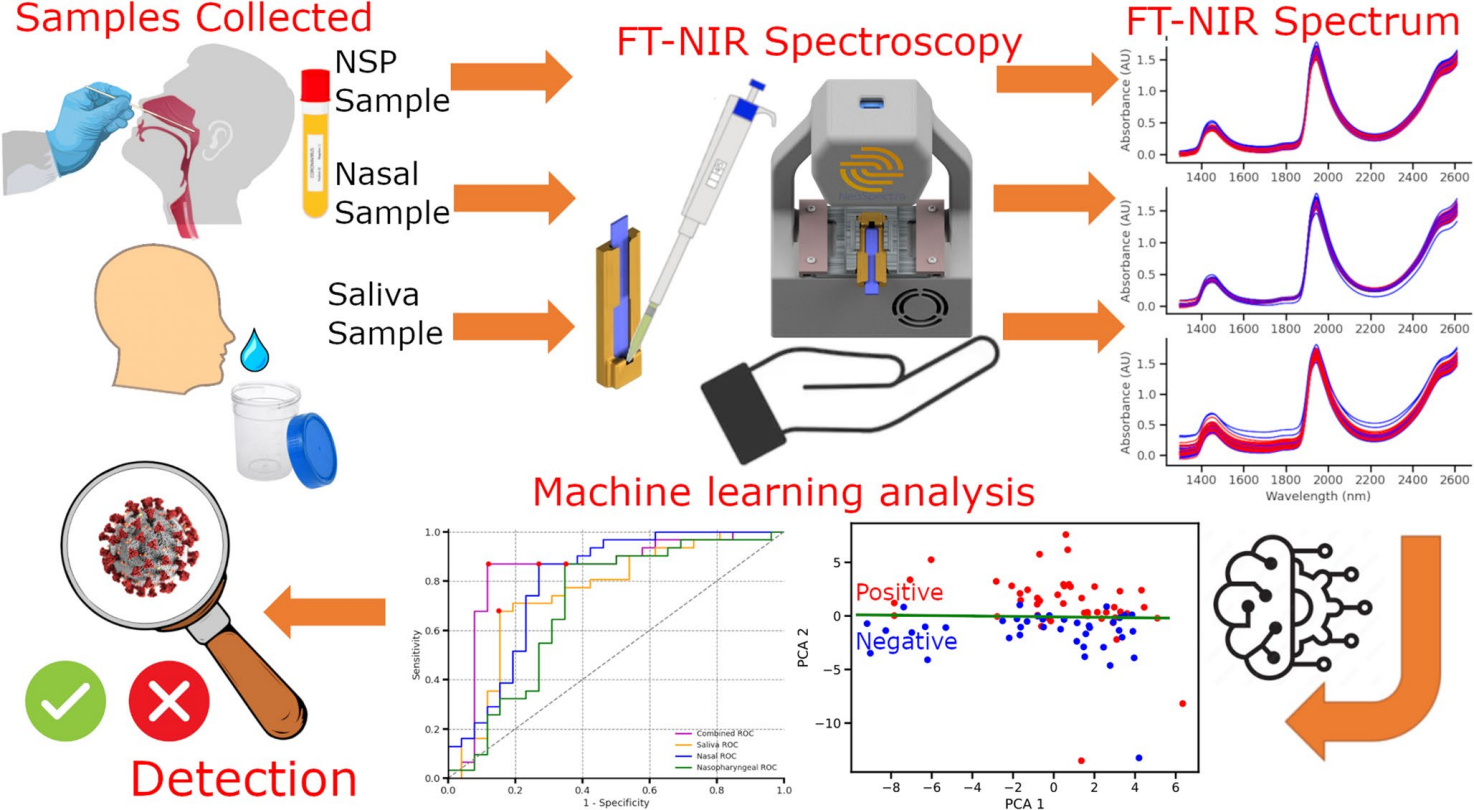
Measurement and Analysis steps for COVID-19 detection. The samples are injected into a cuvette then inserted into a near-infrared portable spectrometer. The resulting spectra are used in different machine learning models to detect the disease in different biofluids

**Fig. 2:**
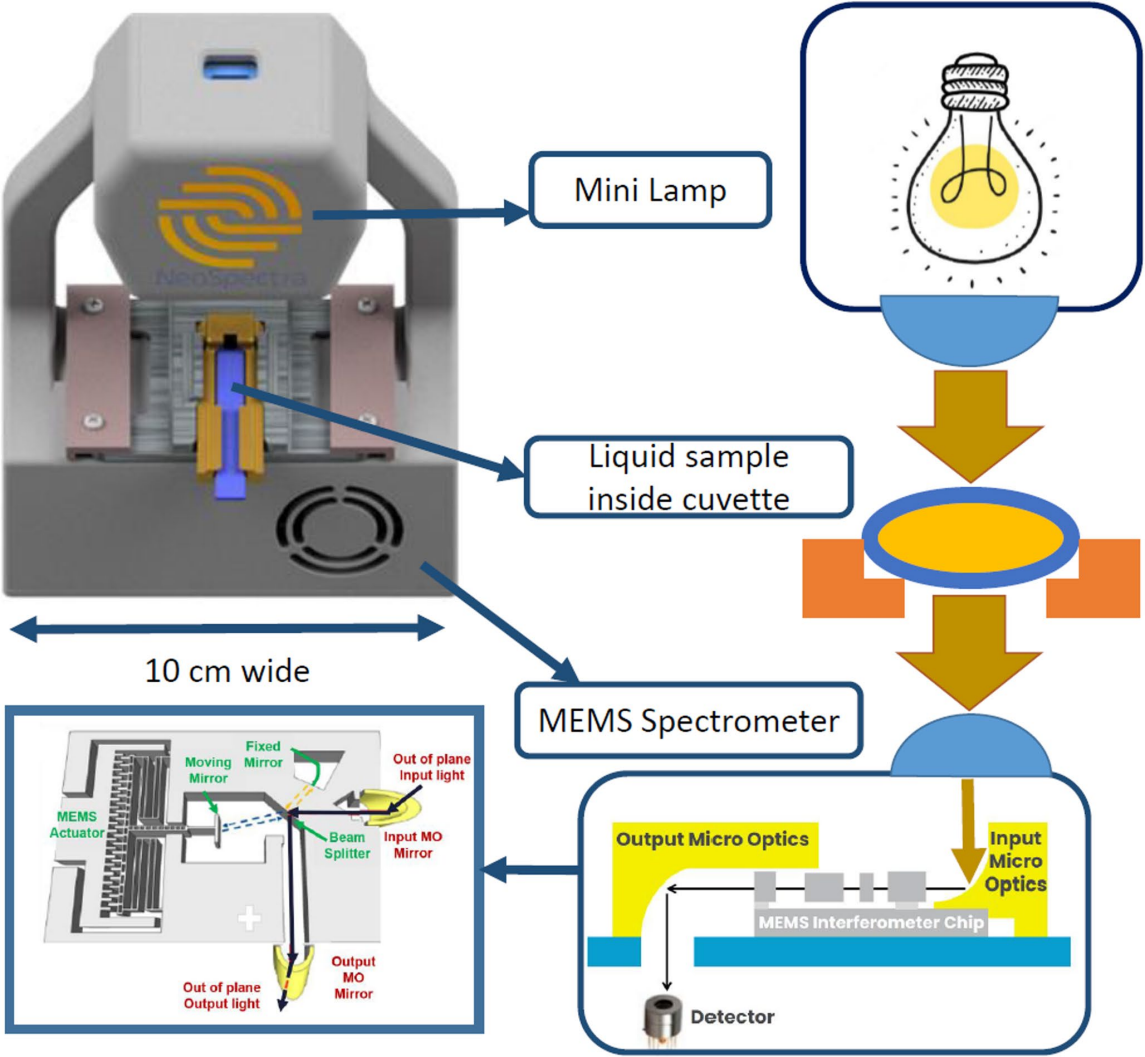
FT-NIR scanner light path. The light radiated from the light bulb is transmitted through the sample then onto a MEMS Michelson interferometer and then to the detector. Light coupling optics are used along the path to maximize the signal

For the FT-NIR spectral measurements, the VTM in which the nasal and nasopharyngeal swabs were stored was identified as the optimal material for scanning. This decision was based on its high homogeneity and the repeatability of its spectral signature. No additional preparation steps were applied to the VTM samples prior to scanning in order to ensure consistency and minimize measurement variance. Similarly, saliva samples were scanned directly without any additives or pre-processing. Consequently, the spectroscopic measurement procedure for a single sample took less than three minutes. This approach supports the primary objective of maintaining a simple, preparation-free workflow to enable classification in POC mass screening applications. The complete measurement process is illustrated in Fig. 1, and a detailed view of the scanning device is provided in Fig. 2. Transmission spectroscopy was used for sample analysis, where the spectrometer analyses the light transmitted through a sample contained in a disposable glass cuvette. This allows a high control on the light path length through the sample and improves repeatability, which further improves the detection accuracy.

### Near-infrared portable measurement system characterization

**Fig. 3:**
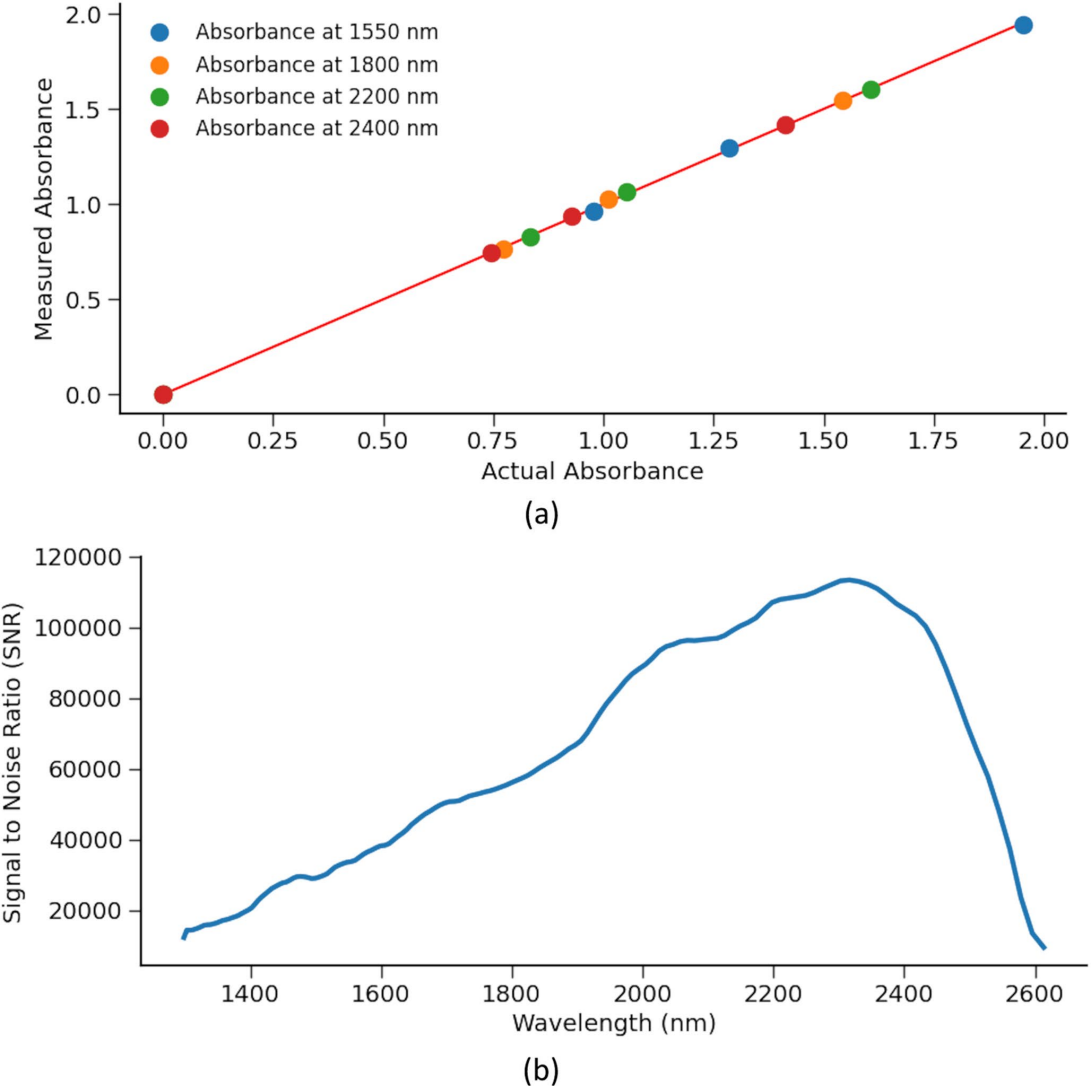
MEMS spectrometer (**a**) linearity and (**b**) signal to noise ratio

Transmission spectroscopy in the NIR region requires a highly repeatable and robust measurement system, as this spectral range captures only the overtones of molecular vibrations. As a result, the system must meet stringent requirements for signal-to-noise ratio (SNR) and measurement consistency. The measurement system composed of an in-house MEMS-based free-space coupled spectrometer integrated with an NIR extended InGaAs detector in a 1 cm^3^ package, which allows the miniaturization of the measurement setup. The details of the spectrometer are discussed in a previous work (Sabry et al. 2015) with the illustrative schematic shown in Fig. 2. The small size of the spectrometer limited the attainable resolution to 66 cm^− 1^, (16 nm at 1550 nm), and the measurable wavelength range is limited to 1.3–2.6 μm. In order to have a robust system that is immune to vibrations and shielded from interference, a metallic housing was fabricated to enclose the spectrometer and the associated electronics along with the light source and a fixture for the cuvette in pre-aligned manner. The small size of the spectrometer allowed the miniaturization of the measurement system to be compact and handheld, with the total size of the measurement system being 5 × 10 × 13 cm^3^, as shown in Fig. 2. The housing allows the device to rest vertically on a table during sample measurement, where the light source is positioned above the sample and the spectrometer. In order to verify the feasibility of the system for the application at hand, the system SNR of a 1 min measurement time as well as the system linearity are independently measured and presented in Fig. 3. The linearity was measured using neutral density filters (Thorlabs).

**Fig. 4:**
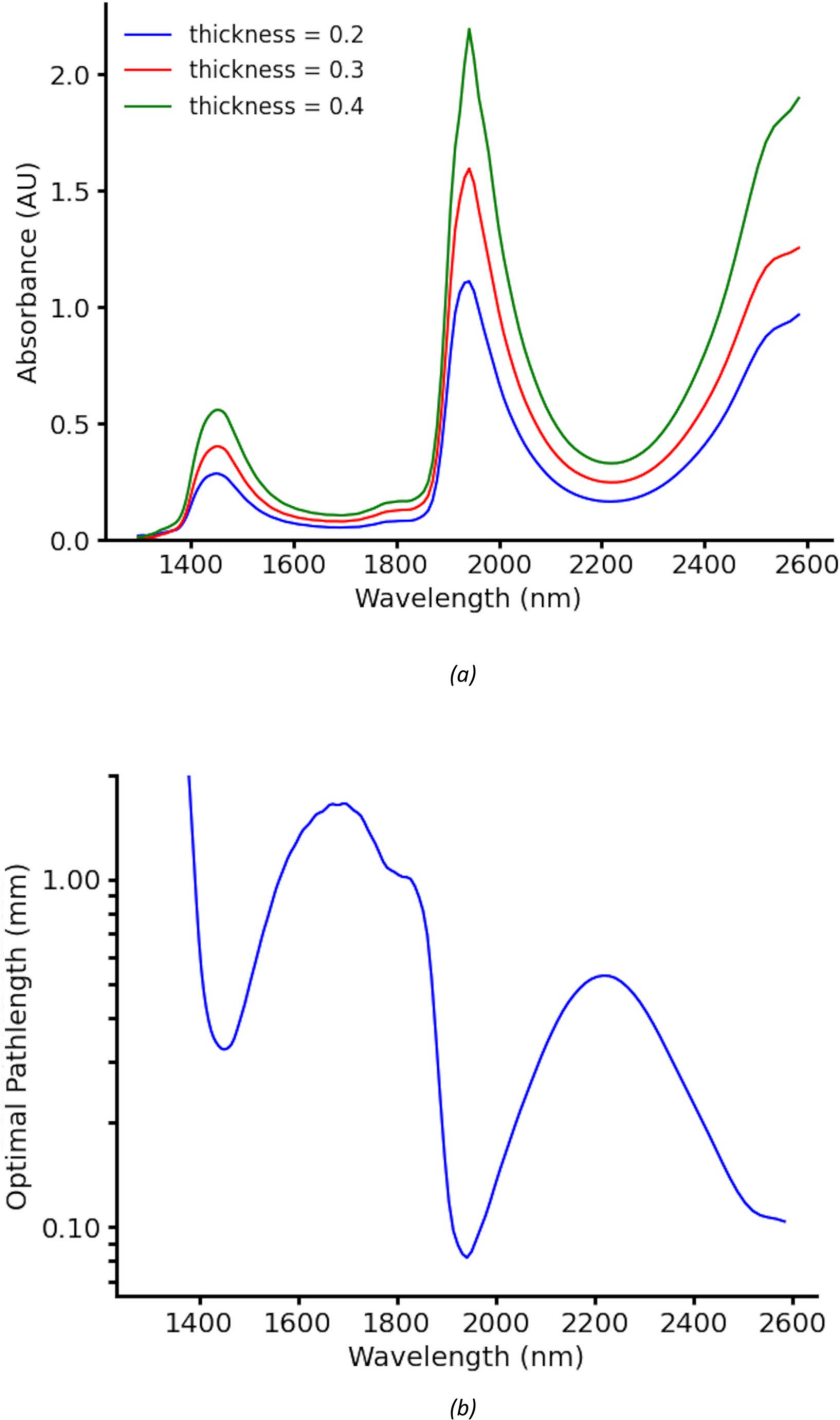
Determination of optimal path length. (**a**) Measurements of VTM at different path lengths. (**b**) Calculated path length required for an absorbance of 0.4343 from VTM

Transmission spectroscopy was performed by sampling 45 µL of VTM or saliva using a pipette and injecting it into a thin-walled cuvette (Vitrocom) mounted in a 3D-printed disposable holder. To determine the optimal optical pathlength for the cuvette, the absorbance of VTM was measured at multiple path lengths, as illustrated in Fig. 4 (a). Following the approach proposed by Jensen et al. (2002) and applying the Beer-Lambert Law, the optimal pathlength corresponds to an absorbance value of 0.4343, which ensures a balance between sensitivity and dynamic range. This relationship is shown in Fig. 4 (b). Based on this analysis, a pathlength of 0.3 mm was selected as the optimal value. This choice maximizes the signal-to-noise ratio across the full spectral range while avoiding saturation effects near the strong water absorption peak at 1940 nm.

After the glass cuvette is placed into a disposable 3D-printed holder, the cuvette is inserted into a fixture positioned between the light source and the spectrometer. Each sample is scanned for one minute to maximize the SNR. Prior to sample insertion, the spectrum of the empty cuvette is also recorded over a one-minute interval to serve as the background reference for subsequent measurements. After each measurement, the cuvette and its holder are disposed of in a chemical waste container to eliminate any risk of cross-contamination. Additionally, the system is cleaned with alcohol and a cotton swab before each new measurement to ensure hygiene and measurement integrity. The entire spectroscopic measurement process takes an average of 3 min per sample.

**Fig. 5:**
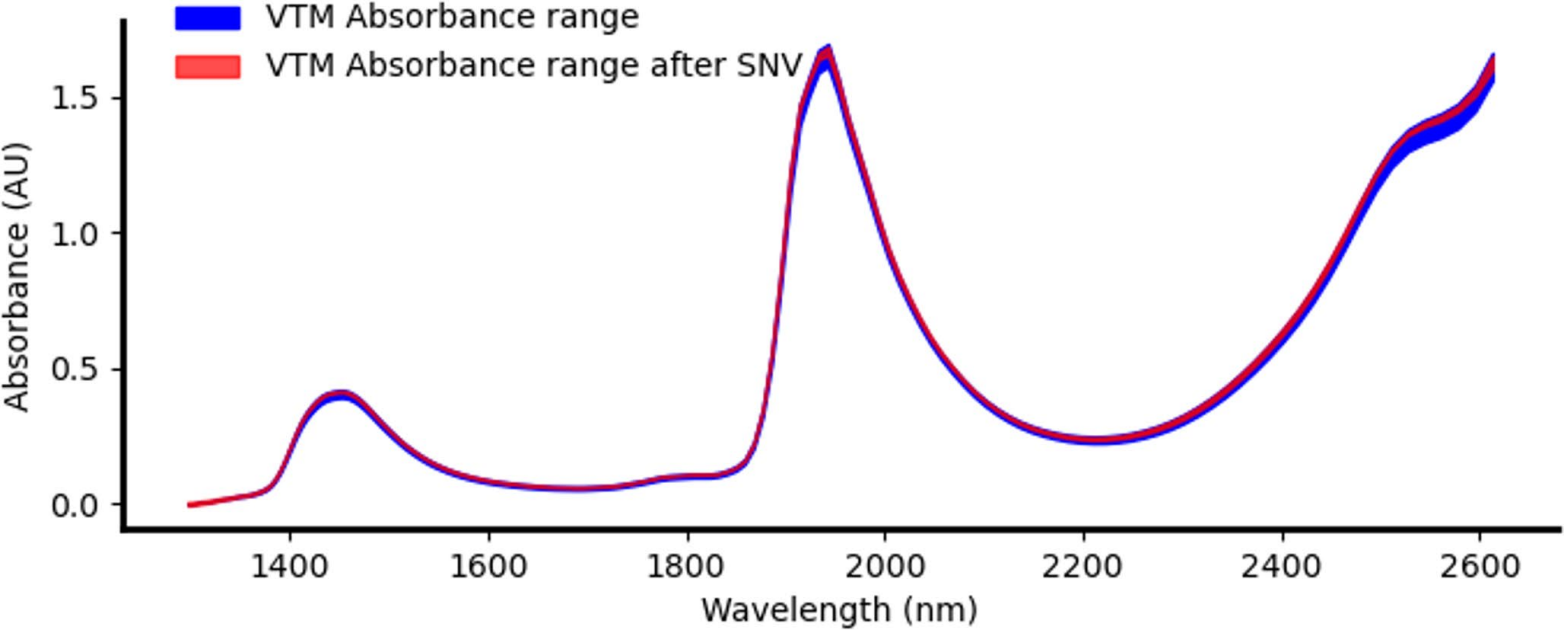
Absorbance range of 10 untainted VTM samples, showing the variance introduced by different cuvettes and holders. The variability is primarily due to differences in light interaction path length, caused by manufacturing tolerances in the cuvettes. The effect of applying Standard Normal Variate (SNV) preprocessing is also shown, demonstrating a significant reduction in spectral variance

To assess the robustness of the measurement system, the variance caused by differences in cuvettes and VTM absorbance was evaluated. Figure 5 shows the maximum deviations in the spectrum of pure VTM when measured using different cuvettes and holders. Variations in path length are most noticeable around the water absorbance peaks, which remained within the manufacturer’s specified tolerances. This variability can be significantly reduced by applying pre-processing techniques such as multiplicative scatter correction (MSC) and standard normal variate (SNV) (Jiao et al. 2020). Both methods were tested as part of the pre-processing workflow, and the effect of SNV on the maximum deviation observed in measurements of 10 untainted VTM samples is also presented in Fig. 5. These techniques are known to reduce device-related variability without altering the intrinsic features of the samples.

### Data processing and analysis

**Fig. 6:**
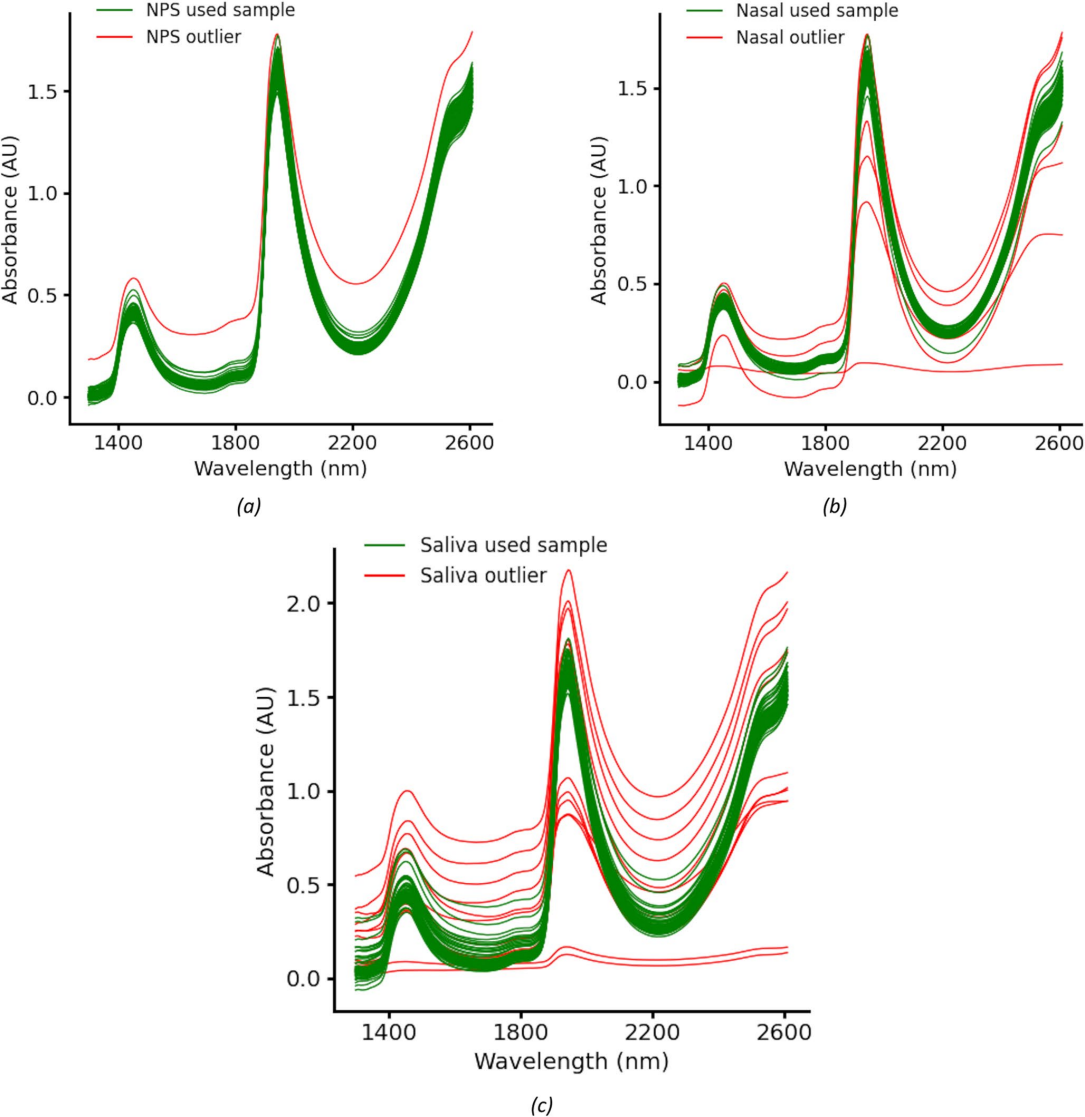
Outlier spectra identified for (**a**) nasopharyngeal, (**b**) nasal, and (**c**) saliva samples. Most outliers are attributed to the presence of air bubbles in the samples, which distort the spectral readings. Saliva samples exhibit the highest number of outliers due to a greater prevalence of air bubbles

A total of 93 patients consented to participate in the study, including 53 COVID-positive and 40 COVID-negative individuals. Among the collected spectra from patients, 1 nasopharyngeal spectra and 7 nasal spectra and 13 saliva spectra were identified as outliers and removed using the Isolation Forest algorithm (Liu et al. 2008). These outlier spectra are shown in Fig. 6. The primary causes of these anomalies include the presence of mucus and air bubbles in the samples, as well as the limitations discussed earlier in the sample collection section, particularly those affecting the quality and quantity of saliva samples.

Data preprocessing and analysis were conducted using the Scikit-learn machine learning package in Python. Prior to modeling, several preprocessing steps were applied to the spectral data, including multiplicative scatter correction and first-order baseline removal. The modeling approach was based on interval Partial Least Squares Discriminant Analysis (iPLS-DA) (Nørgaard et al. 2000). PLS analysis identifies components in the data that are correlated with the output variable, thereby reducing dimensionality. A linear regression model is then applied to these components, and a classification threshold is selected using cross-validation.

iPLS-DA divides the spectral range into 10 intervals and constructs a separate PLS-DA model for each interval, as well as combinations of intervals, based on their classification accuracy. The optimal combination of spectral regions and the number of components is determined through cross-validation. Cross-validation was performed using the One-Patient-Out Cross-Validation (OPOCV) method. In each iteration, the model is trained on all samples except one, which is used for testing. This process is repeated until every sample has been used once as the test set. The results from all iterations are then aggregated to determine the optimal classification threshold, evaluate model performance, and select the most effective preprocessing strategy. This allows us to investigate the bands with the highest accuracy for virus detection as well as the accuracy of such region.

In order to test the stability of this method and obtain a lower bound on the device accuracy when tested on future clinical trials, nested cross-validation is done using stratified K-Fold where the dataset is first split into 10 parts. 1 part is used as a test set and the other 9 parts are used for model creation and parameter selection using an inner One-Patient-Out Cross-Validation method. This process is repeated using each of the 10 parts as the test set, resulting in different model parameters being used for each part of the dataset. Then, the result of the 10 parts being used as test set is aggregated for the final performance metrics.

To reduce variability in cycle threshold (Ct) values across different sampling methods, a separate set of models was built using only data from patients who had viable spectra for all three sampling types. Additionally, to evaluate the added benefit of combining data from all three sample types—nasal swabs, nasopharyngeal swabs, and saliva—an integrated analysis was performed using the concatenated spectra from each patient’s three samples.

## Results and discussion

**Fig. 7:**
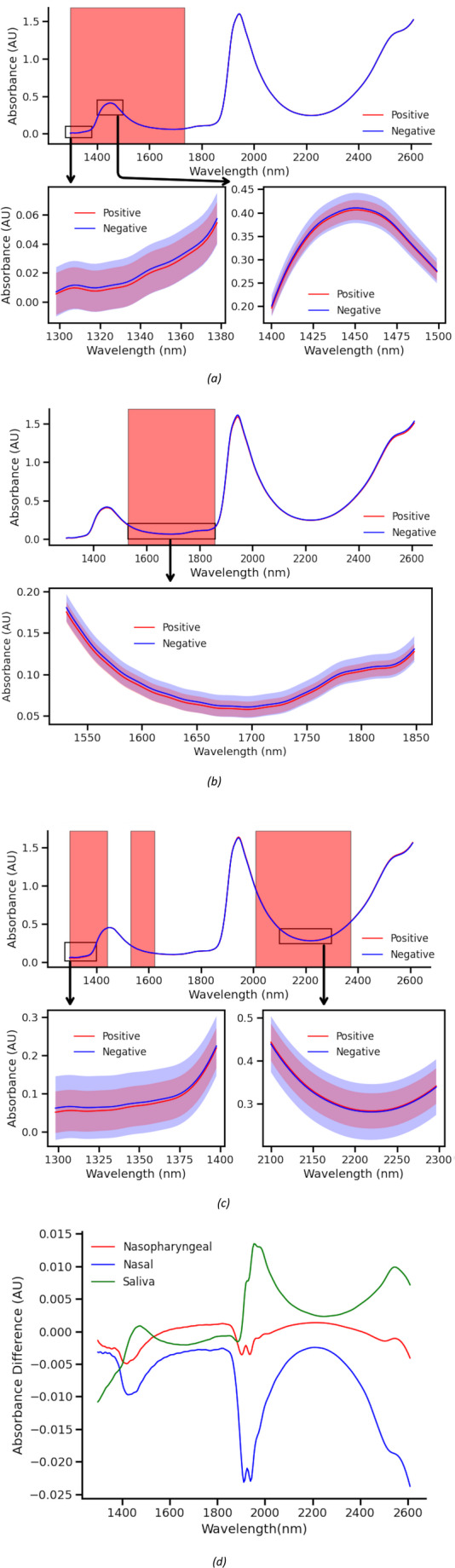
Average spectra for the different sample types, with the mean represented by the solid line and the standard deviation indicated by the shaded area. Spectral regions selected by the iPLS algorithm for highest classification accuracy are highlighted in red. (**a**) For nasopharyngeal samples, the OH first overtone region demonstrates the highest predictive power. (**b**) For nasal samples, the CH first overtone region is most informative. (**c**) For saliva samples, both the OH first overtone region and the CH combination band region contribute most to model accuracy. (**d**) Difference spectrum between positive and negative samples

Figure 7 presents the average spectra of COVID-positive and COVID-negative samples for each sampling method, with the regions selected by the iPLS algorithm highlighted. A key region identified across all three models is the C–H first overtone region (1500–1800 nm), which strongly indicates the presence of biological materials in the scanned samples (Beć et al. 2020). For the nasopharyngeal model, the most informative spectral features extend into the C–H second overtone region (1300–1500 nm), contributing to improved model accuracy. In the case of saliva samples, in addition to the first and second C–H overtone regions, the combination band region (2000–2500 nm) also plays a significant role in enhancing model performance.

**Fig. 8:**
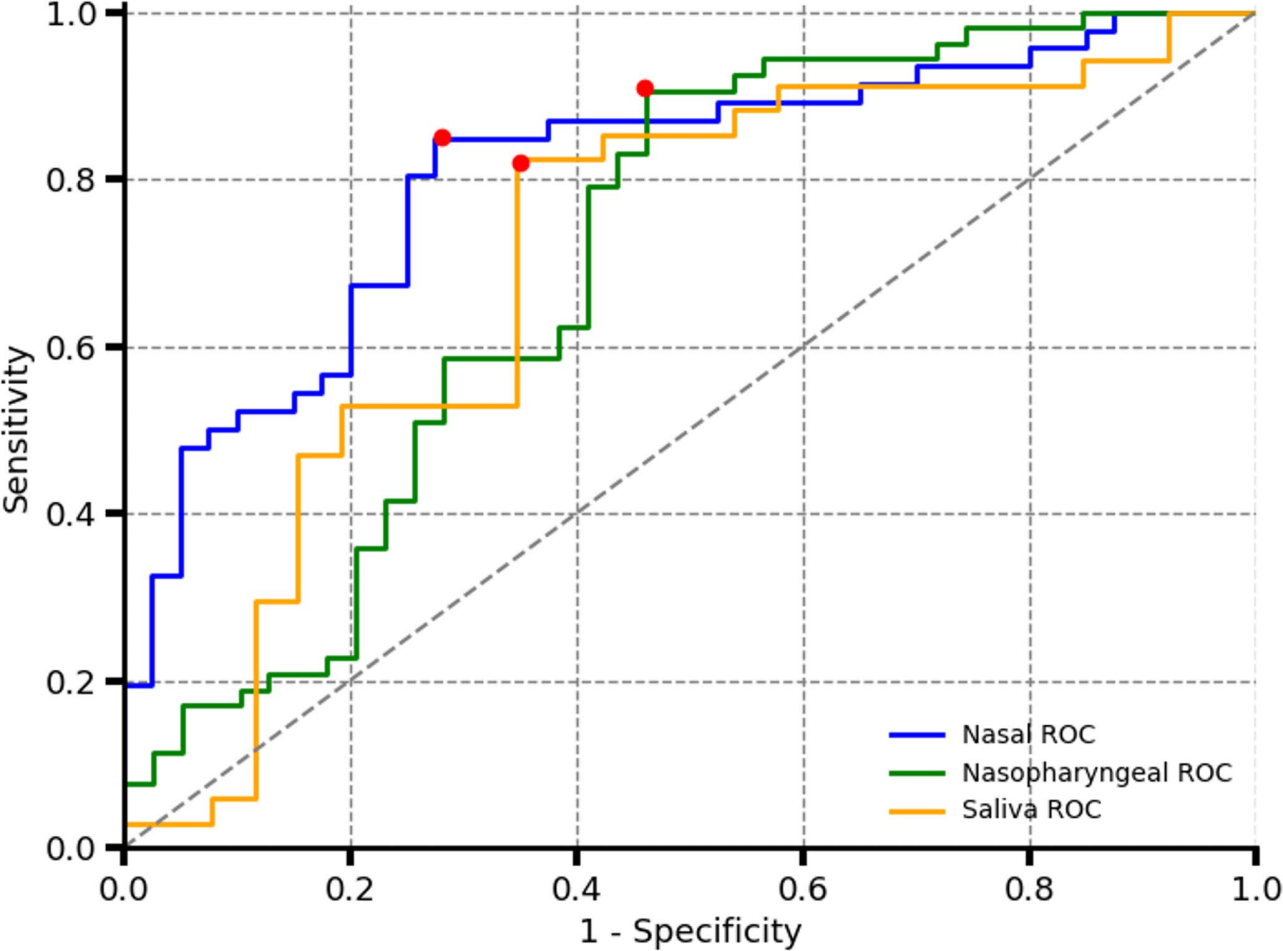
Receiver operating characteristic (ROC) curves for all collected sample types. The red markers indicate the points of highest accuracy, as summarized in Table 1. Nasal samples exhibit the highest overall accuracy and specificity for the disease detection, whereas nasopharyngeal samples demonstrate the highest sensitivity

Figure 8 displays the Receiver Operating Characteristic (ROC) curves for the models built using the collected data, with the corresponding optimal performance points summarized in Table 1. All three sampling techniques demonstrate comparable performance in predicting COVID-19 status, with the nasal swab model achieving the highest accuracy at 79% ± 8.4% (95% confidence interval). The average Ct value for the true positive and false negative samples are shown in Table 2. A lower average Ct value for the true positive classification indicates a higher capability at classifying patients with a higher viral load. Table 3 shows the results of using nested cross-validation to estimate the device performance using multiple models.

**Table 1:** iPLS cross-validation model performance for all collected data

Samples Type	Accuracy ± 95% CI	Sensitivity TP/(TP + FN)	Specificity TN/(TN + FP)	False Positive Rate FP/(TN + FP)	False Negative Rate FN/(TP + FN)	AUC	Positive Samples	Negative Samples
Nasopharyngeal	75% ± 9.8%	91%	54%	46%	9%	0.7	53	39
Nasal	79% ± 8.4%	85%	72%	28%	15%	0.81	46	40
Saliva	75% ± 10.8%	82%	65%	35%	18%	0.71	34	26

**Table 2:** Correlation between Ct value and iPLS cross-validation model performance

Samples Type	True Positive Classifications Average Ct value	False Negative Classifications Average Ct Value
Nasopharyngeal	24.0	29.7
Nasal	27.0	29.1
Saliva	27.8	29.7

**Table 3:** iPLS models average performance using nested cross-validation

Samples Type	Accuracy ± 95% CI	Sensitivity TP/(TP + FN)	Specificity TN/(TN + FP)	False Positive Rate FP/(TN + FP)	False Negative Rate FN/(TP + FN)	AUC	Positive Samples	Negative Samples
Nasopharyngeal	64% ± 8.5%	79%	43%	57%	21%	0.61	53	39
Nasal	67% ± 9.4%	72%	63%	37%	28%	0.67	46	40
Saliva	66% ± 10.4%	76%	53%	47%	24%	0.61	34	26

Both nasopharyngeal and nasal samples exhibit high sensitivity of 91% and 85% respectively; this comes at the cost of reduced specificity. Such characteristics make these sampling methods suitable for large-scale initial screening, where positive cases can subsequently be confirmed using the slower and more expensive PCR tests. Saliva-based models, on the other hand, offer more balanced performance, with a sensitivity of 75% and specificity of 82%. These results are consistent with findings reported by Khiabani (2021), which indicate that nasopharyngeal swabs generally yield higher sensitivity than saliva samples in COVID-19 detection.

An accuracy of 75% − 79% for NIR spectroscopy is comparable to other POC testing methods, such as rapid antigen test kits, which were reported to have an accuracy of 77% − 92% (Lam et al. 2026), and serology tests having 53%−97% accuracy depending on the time of infection (Leony et al. 2024).

**Fig. 9:**
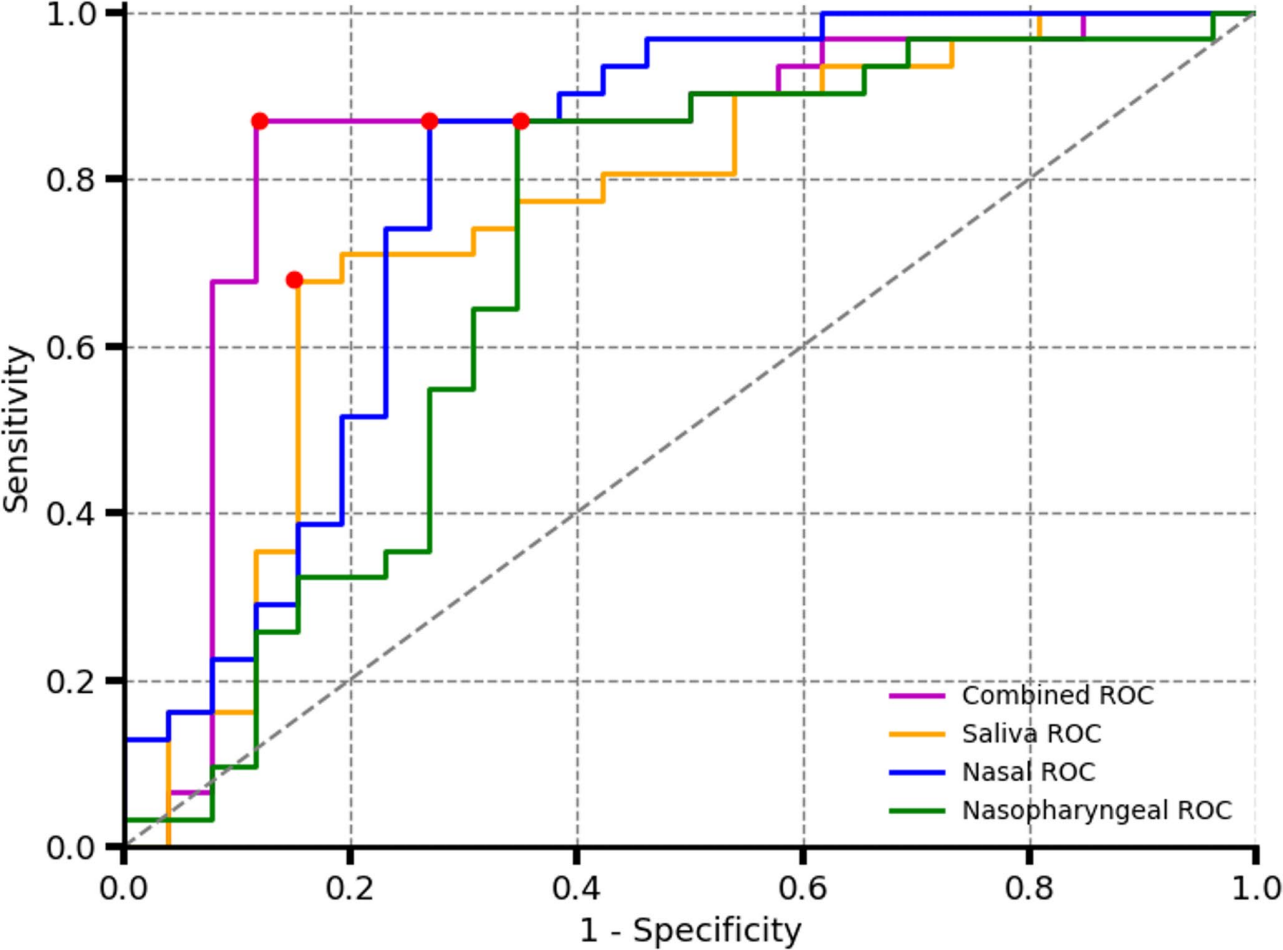
Receiver operating characteristic (ROC) curves for patients with all three sample types (nasal, nasopharyngeal, and saliva). Red markers indicate the points of highest accuracy, as detailed in Table 2. Combining the spectra from all three sample types results in improved overall detection accuracy

Table 4 presents the performance of models built using spectra from the same patients across all sampling methods. Among the individual models, the nasal sample model achieved the highest accuracy at 80% (95% CI: 70%–90%) for the given sample size. As shown in the ROC curves in Fig. 9, the nasal model also demonstrates the highest sensitivity across a broader portion of the curve compared to the other sampling methods, followed closely by the nasopharyngeal model. However, the differences between the three individual models remain relatively small.

**Table 4:** iPLS cross-validation model performance for patients with valid spectrum for the 3 scanned fluids

Samples Type	Accuracy ± 95% CI	Sensitivity TP/(TP + FN)	Specificity TN/(TN + FP)	False Positive Rate FP/(TN + FP)	False Negative Rate FN/(TP + FN)	AUC	Positive Samples	Negative Samples
Nasopharyngeal	77% ± 11.6%	87%	65%	35%	13%	0.71	31	26
Nasal	80% ± 10.2%	87%	73%	27%	13%	0.80	31	26
Saliva	76% ± 10.3%	68%	85%	15%	32%	0.76	31	26
Combined model	88% ± 8.4%	87%	88%	12%	13%	0.85	31	26

Most notably, the model constructed using the combined spectra from all three sample types achieved a significantly improved accuracy of 88% (95% CI: 80%–96%). While this enhancement highlights the benefit of data fusion, it comes at the cost of requiring all three samples from each patient, which triples the sampling and scanning effort. Nevertheless, this trade-off may be acceptable in high-throughput POC or mass screening settings, particularly given the portability and low cost of the proposed MEMS-based device. Table 5 shows the results of using nested cross-validation to estimate the device performance using multiple models, which indicates the same trend.

**Table 5:** iPLS models average performance using nested cross-validation for patients with valid spectrum for the 3 scanned fluids

Samples Type	Accuracy ± 95% CI	Sensitivity TP/(TP + FN)	Specificity TN/(TN + FP)	False Positive Rate FP/(TN + FP)	False Negative Rate FN/(TP + FN)	AUC	Positive Samples	Negative Samples
Nasopharyngeal	66% ± 11.6%	67%	65%	35%	33%	0.69	31	26
Nasal	67% ± 10.2%	54%	80%	20%	46%	0.64	31	26
Saliva	65% ± 10.3%	58%	73%	27%	42%	0.6	31	26
Combined model	77% ± 9.8%	83%	69%	31%	17%	0.76	31	26

Although the nasopharyngeal swab method has consistently demonstrated the highest diagnostic accuracy for detecting infected patients in prior studies using reference methods, the results of this study suggest that nasal, nasopharyngeal, and saliva samples exhibit comparable predictive power when analyzed using NIR spectroscopy. This discrepancy highlights the possibility that NIR spectra capture features correlated with the presence of the virus, but not necessarily with the viral load itself. Instead, the spectral signal may reflect other biological components present in the samples, such as mucus and various nasal and oral fluids. This observation warrants further investigation into the specific spectral markers associated with SARS-CoV-2 and the physiological changes induced by infection.

It is also important to note that all patients were already admitted to the isolation hospital at the time of sample collection and were exhibiting symptoms of COVID-19. This may have influenced the spectral data and overall results, and should be considered as a potential limitation of the study.

## Conclusion

Near-infrared optical spectroscopy using portable scanners presents a promising tool for the detection of COVID-19 by analyzing the light transmitted through nasal or nasopharyngeal swab VTM or saliva samples. This study establishes an upper bound of 80% accuracy (95% CI: 70%–90%) for the disease detection using nasal swab samples analyzed with NIR spectroscopy. The classification performance improves to 88% accuracy when spectra from multiple sample types nasopharyngeal, nasal, and saliva are combined. However, this comes at the cost of increased sampling and scanning complexity. The achieved results combined with the low cost and portability of NIR spectroscopy make it a viable candidate for point-of-care diagnostics and initial mass screening, particularly in resource-limited settings. The detection threshold can be tweaked towards higher sensitivity to be leveraged for screening large populations, with positive results subsequently confirmed via more specific methods like PCR, thereby reducing the overall cost and logistical burden of mass testing programs. This approach is especially valuable in developing countries where PCR infrastructure may be limited or unavailable.

## Data Availability

The data that support the findings of this study are available from the corresponding author upon reasonable request.
